# Gray matter reduction in bilateral insula mediating adverse psychiatric effects of body mass index in schizophrenia

**DOI:** 10.1186/s12888-022-04285-4

**Published:** 2022-10-11

**Authors:** Hui Wu, Guochao Dai, Muyeseer Aizezi, Juan Tang, Ke Zou, Yuhua Wu, Xiaoli Wu

**Affiliations:** 1 Psychiatry Department, The Third Affiliated Hospital of Sun Yat-Sen University, 600 Tianhe Road, Tianhe District, Guangzhou, Guangdong China; 2 The Affiliated Kashi Hospital of Sun Yat-Sen University, Kashi, China; 3 Radiology Department, The First People’s Hospital of Kashi Prefecture, Kashi, China; 4 Psychiatry Department, The First People’s Hospital of Kashi Prefecture, 120 Yingbin Avenue, Kashi, Xinjiang China

**Keywords:** Schizophrenia, Obesity, Gray matter, Insula, Negative symptom

## Abstract

**Background:**

Both schizophrenia (SZ) and overweight/obesity (OWB) have shown some structural alterations in similar brain regions. As higher body mass index (BMI) often contributes to worse psychiatric outcomes in SZ, this study was designed to examine the effects of OWB on gray matter volume (GMV) in patients with SZ.

**Methods:**

Two hundred fifty subjects were included and stratified into four groups (*n* = 69, SZ patients with OWB, SZ-OWB; *n* = 74, SZ patients with normal weight, SZ-NW; *n* = 54, healthy controls with OWB, HC-OWB; and *n* = 53, HC with NW, HC-NW). All participants were scanned using high-resolution T1-weighted sequence. The whole-brain voxel-based morphometry was applied to examine the GMV alterations, and a 2 × 2 full factorial analysis of variance was performed to identify the main effects of diagnosis (SZ vs HC), BMI (NW vs OWB) factors, and their interactions. Further, the post hoc analysis was conducted to compare the pairwise differences in GMV alterations.

**Results:**

The main effects of diagnosis were located in right hippocampus, bilateral insula, rectus, median cingulate/paracingulate gyri and thalamus (SZ < HC); while the main effects of BMI were displayed in right amygdala, left hippocampus, bilateral insula, left lingual gyrus, and right superior temporal gyrus (OWB < NW). There were no significant diagnosis-by-BMI interaction effects in the present study, but the results showed that both SZ and OWB were additively associated with lower GMV in bilateral insula. Moreover, mediation analyses revealed the indirect effect of BMI on negative symptom via GMV reduction in bilateral insula.

**Conclusion:**

This study further supports that higher BMI is associated with lower GMV, which may increase the risk of unfavourable disease courses in SZ.

**Supplementary Information:**

The online version contains supplementary material available at 10.1186/s12888-022-04285-4.

## Introduction

Schizophrenia (SZ) is a highly disabling mental disorder with a lifetime prevalence of approximately 1% [1], imposing an enormous burden on public health. Until now, the pathogenesis and development of SZ remains unelucidated, and current treatment approaches supported by evidence-based medicine are limited [2]. Hence, it is important to clarify the etiology and neurobiological mechanisms of SZ from a more in-depth perspective.

With the development of magnetic resonance imaging (MRI) technology, researchers have identified multiple brain structural alterations associated with SZ, the most commonly observed of which are the frontal, temporal, insular, and mesostriatal brain regions [3, 4]. Nevertheless, the location and direction of these brain regions often vary across studies, and the reasons for this heterogeneity remain unknown. They may be related to premorbid neurodevelopmental differences or the presence of certain clinical characteristics [5]. As such, a better understanding of the impacts of key clinical factors on brain alterations is critical for interpreting these inconsistent findings, and it is also the first step towards their management. Of note, excess body weight, such as being overweight or obese, may be a potential source of heterogeneity in brain structural alterations.

Clinically, overweight/obesity is particularly prevalent in SZ, with nearly 40–60% of patients with SZ being overweight or obese, a rate significantly higher than that in the general population (20–40%) [6–9]. Among the factors contributing to this high prevalence, a complex interplay between the adverse effects of antipsychotics, unhealthy lifestyle, and pathophysiological mechanisms inherent in SZ, have been the most cited explanations [7]. Notably, SZ patients with overweight/obesity tend to have poorer cognitive performance and physical condition, as well as shorter life expectancy than their lean counterparts [10]. In light of this, weight gain abnormalities are also considered one of the strongest contributors to adverse psychiatric outcomes in SZ [11]. In addition to these observations, researchers have also demonstrated that being overweight or obese is related to altered brain structure in the general population [12]. Specifically, overweight/obesity-related brain structural alterations have been identified in a wide range of areas involved in emotion and memory (e.g., the amygdala, hippocampus), cognitive control (e.g., cingulate, prefrontal cortex), reward (e.g., the striatum, insula, orbitofrontal cortex), and regulation of energy homeostasis (e.g., the hypothalamus) [13–15]. Notably, all these brain regions, as well as the associated neural circuits and functions are frequently disrupted in SZ [16]. Indeed, obesity has been suggested to share some common pathophysiological mechanisms with various mental disorders, including SZ [17, 18]. Overlapping pathoetiologies include chronic low-grade inflammation, increased oxidative stress, neurotransmitter imbalances, and hypothalamic–pituitary–adrenal axis disturbances [17, 18], which might be related to the common neuroimaging abnormalities and high prevalence of obesity in patients with SZ.

As aforementioned, both SZ and overweight/obesity showed structural alterations in similar brain regions. Since the prevalence of overweight/obesity is remarkably increased in patients with SZ, previous findings on brain structural changes in SZ may have been confounded by the effects of weight gain abnormalities. Accordingly, future study investigating brain morphometric alterations in SZ should carefully stratify the groups according to BMI. Moreover, comorbidity with overweight/obesity often contributes to an unfavorable course of disease [6, 19], suggesting that similar brain structural changes might have mediated the adverse influence of overweight/obesity on clinical outcomes in SZ. Thus, the relationship between BMI, brain structural alterations, and clinical symptoms should be clarified. Therefore, in the present study, we performed (1) a 2 × 2 full factorial analysis of variance (ANOVA) to examine the effects of diagnosis, BMI, and their interactions on GMV using a whole-brain voxel-based morphometry (VBM) approach, (2) a post hoc analysis to determine the pairwise comparison in brain regions with significant GMV alterations, and (3) association and mediation analyses to investigate the underlying relationship among BMI, GMV alterations, and clinical symptoms.

To our knowledge, while the effects of overweight/obesity on brain structure are of great concern in SZ, few studies have adopted such a design to examine overweight/obesity-related brain structural alterations and whether these directly overlap with brain regions associated with SZ [20]. Therefore, our research may address this knowledge gap. We hypothesized that both SZ and overweight/obesity will be negatively related to some GMV alterations in similar brain regions, and that the relationship between BMI and clinical symptoms may be mediated by GMV alterations.

## Methods

### Participants

In total, 250 Chinese Uyghur participants were included into this study, including 69 SZ patients with overweight or obesity (SZ-OWB), 74 SZ patients with normal weight (SZ-NW), 54 healthy controls with OWB (HC-OWB), and 53 healthy controls with NW (HC-NW). The healthy participants were recruited from physical examination center and local communities through advertising. The SZ patients were recruited from the Psychiatry Department of the Affiliated Kashi Hospital of Sun Yat-Sen University, Kashi, Xinjiang, China. The BMI category are as follows: normal weight as 18.50 ≤ BMI < 25.00 kg/m^2^, overweight or obesity as BMI ≥ 25.00 kg/m [21].

Inclusion criteria for SZ patients: (1) met the diagnostic criteria for SZ according to the International Classification of Disease 10th Revision (ICD-10); (2) age between 18 and 55; (3) right handedness; (4) had been educated for at least six years.

Inclusion criteria for HC: (1) no personal or family history of mental disorder refer to mini-SCID; (2) age between 18 and 55; (3) right handedness; (4) had been educated for at least six years.

Exclusion criteria: (1) history of other mental disorder, including anxiety, depression, bipolar disorder, mental retardation, eating disorders, etc.; (2) significant brain lesions, including cerebral infarction, hemorrhage, or intracranial masses; (3) alcohol or substance addiction; (4) metabolic related diseases such as hypertension, diabetes, hypothyroidism or hyperthyroidism; (5) any MRI contraindications, such as metallic implants and claustrophobia; (6) pregnant or lactating women.

### Psychological and clinical assessments

The SZ was diagnosed by a chief psychiatrist based on the ICD-10 diagnostic criteria [22]. The positive and negative syndrome scale (PANSS) was used to quantify the severity of symptoms in SZ patients. The Montreal cognitive assessment (MoCA) was adopted to measure the cognitive performance in all participants. The MoCA is a cognitive screening test, which provided scores for seven cognitive domains: visuospatial/constructional abilities, naming, language, attention, abstraction, delayed memory, orientation [23].

Additionally, all the participants were asked to fill in a designed questionnaire, including sex, age, ethnicity, education background, duration of illness, age of onset, number of hospitalizations, antipsychotics history (type, dose and duration), and lifestyle (diet, smoking, and drinking habits). The body parameters including height, weight as well as waist and hip circumferences of all participants were measured and recorded by the same nurse. All the psychological and clinical assessments were conducted within three days after MRI scanning.

### Imaging acquisition

The MRI data was obtained on 3.0 T magnetic resonance scanner (Magnetom Skyra, Siemens, Erlangen, Germany) equipped with a 32-channel head coil. Participants lied on the scanner in the supine position with eyes closed, and foam pads were used to minimize head motion. The high-resolution sagittal T1-weighted images were acquired using a T1-weighted magnetization-prepared-rapid-acquisition-gradient-echo (MP-RAGE) sequence (repetition time = 2000.0 ms, echo time = 2.22 ms, inversion time = 900 ms, flip angle = 8°, field of view = 256 × 256 mm^2^, voxel size = 0.9 × 0.9 × 0.9 mm^3^, and 176 slices). The total scan time was 3 min and 54 s.

### Voxel-based morphometry (VBM) analysis

The structural data were preprocessed using optimized VBM8 toolbox (http://dbm.neuro.uni-jena.de/vbm/) for SPM8 (http://www.fil.ion.ucl.ac.uk/spm) under a MATLAB (version R2013b, MathWorks, MA, USA) environment. Briefly, the main steps include: a) discarded the images with visible artifacts; b) format conversion and corrected the image origin (reorientation); c) bias field correction; d) structural images were segmented into three tissue components: gray matter (GM), white matter (WM), and cerebrospinal fluid (CSF); e) the segmented images of all subjects were normalized to the Montreal Neurological Institute (MNI) space using the DARTEL deformations (resliced into 1.5mm^3^); f) the resulting GM images ( modulated GMV) were smoothed using an isotropic Gaussian kernel of 8 mm FWHM.

At the secondary level of analysis, we applied a whole-brain voxel-wise general linear model with a 2 (diagnosis: SZ vs HC) × 2 (BMI category: OWB vs NW) full factorial ANOVA, controlling for age, sex, education and total intracranial volume (ICV), to determine the main effects of diagnosis and BMI factors, as well as their interaction effects. The voxel-wise threshold was set at *p* < 0.001, and corrected by a cluster-level Familywise error (FWE) correction. For reporting purposes, the masks of significant cluster were defined using the Automated Anatomical Labeling (AAL) atlas [24]. Then, the brain regions with significant difference were saved as masks, and the mean GMV values within each significant cluster were extracted for all subjects, For each significant cluster, the region of interest (ROI)-based post hoc analyses was applied to compare the pairwise differences among the four groups (HC-NW vs SZ-NW; HC-OWB vs SZ-OWB; HC-NW vs HC-OWB; SZ-NW vs SZ-OWB) while controlling for age, sex, education and total ICV (*p* < 0.001, FWE corrected).

### Statistical analyses

For the demographic and clinical data, the statistical analysis was performed using the SPSS 25.0 (IBM Corp., Armonk, NY), with a threshold of *p* < 0.05 being considered as statistically significant. The continuous variables were summarized by means ± standard deviations, and analysed by independent t-test or one-way ANOVA. The categorical variables were summarized using frequencies, and analysed by Chi-square test. To further test the “diagnosis × BMI” interaction effects on GMV, we performed the multivariate analysis of covariance with diagnosis and BMI as explanatory variables, and regional GMV as the dependent variables, while controlling for age, sex, education and ICV as covariates of no interest. To increase the sensitivity, we also repeated the analyses with BMI as a continuous variable. Correlation analyses of significant GMV with clinical symptoms and cognitive test scores were conducted using Pearson’s partial correlation coefficient. Age, sex, education, duration of illness, BMI and ICV were included as covariates in the partial correlation analysis within the SZ group. Age, sex, education, BMI and ICV were included as covariates in the partial correlation analysis within the overweight/obese group.

To determine whether the effects of BMI on psychiatric symptoms (PANSS) were mediated by altered GMV, the mediation analysis was conducted by employing a model 4 of the PROCESS software (http://www.processmacro.org) implanted in SPSS, controlling for age, sex, education, ICV and duration of illness. The Bonferroni-correction with q < 0.05 was applied to correct for multiple comparisons, resulting in a statistical threshold of *p* < 0.0167 (0.05/3). The direct effects and total effects were considered significant when the *p* < 0.0167 or the 95% confidence intervals (CI) did not include zero in the 5,000 bootstrap samples corrected. The indirect effects were considered significant when the CI did not include zero in the 5,000 bootstrap samples corrected [25]. The mediation effects exist when the indirect effects were statistically significant.

## Results

### Demographic and clinical comparisons

As shown in Table 1, there were no significant differences in age, sex, education, and smoking and drinking rates among the four groups. The two subgroups of patients with SZ did not show significant differences in duration of illness, age of onset, antipsychotic medication use, and PANSS total and subscale scores. As expected, the patients with SZ showed lower scores in the MoCA total and six subscale scores compared to the HC. Compared with SZ patients with normal weight, patients with overweight/obesity displayed lower scores in attention and delayed memory (Table S1); compared with the HC with normal weight, the HC with overweight/obesity displayed lower score in visuospatial/constructional abilities (Table S2).

**Table 1:** Demographic and clinical characteristics for all participants (*n* = 250)

	**SZ**		**HC**		***F/t/***^*2*^	***p* vaue**
	**OWB (*n* = 69)**	**NW (*n* = 74)**	**OWB (*n* = 54)**	**NW (*n* = 53)**		
**Age** (years)^a^	33.88 ± 8.85	31.86 ± 8.70	33.31 ± 7.91	31.42 ± 7.07	1.248	0.293
**Sex** (male/female)	32/37	31/43	23/31	23/30	0.327	0.955
**Education** (years)	10.28 ± 3.67	10.41 ± 3.30	10.83 ± 3.01	11.55 ± 3.34	1.710	0.165
**BMI** (kg/m^2^)	28.61 ± 2.70	21.67 ± 2.44	28.19 ± 2.62	21.63 ± 2.33	149.234	** < 0.001**
**Age of onset** (years)	26.32 ± 8.92	24.76 ± 7.62	N/A	N/A	1.125	0.263
**Duration of illness** (years)	7.81 ± 5.63	7.10 ± 5.32	N/A	N/A	0.772	0.441
**Antipsychotic medications (%)**	58 (84.1)	57 (77.0)	N/A	N/A	0.719	0.397
CPeq (mg/day)	349.6 ± 170.4	374.7 ± 253.9	N/A	N/A	-0.697	0.487
**Smoking regularly** (%)	26 (37.7)	20 (27.0)	15 (27.8)	11 (20.8)	3.327	0.344
**Drinking regularly** (%)	11 (15.9)	6 (8.1)	7 (13.0)	3 (5.7)	4.166	0.244
**PANSS total scores**	81.62 ± 15.06	78.45 ± 17.91	N/A	N/A	1.144	0.255
Positive symptom	21.38 ± 5.33	21.53 ± 6.10	N/A	N/A	-0.156	0.876
Negative symptom	21.25 ± 5.59	20.26 ± 4.90	N/A	N/A	1.128	0.610
General psychopathology	39.00 ± 10.11	36.66 ± 11.76	N/A	N/A	1.271	0.206
**MoCA total scores**	19.46 ± 5.44	20.26 ± 6.02	24.76 ± 3.06	24.57 ± 3.96	19.477	** < 0.001**
Visuospatial/constructional	2.81 ± 1.53	2.86 ± 1.55	3.22 ± 1.19	3.75 ± 1.37	5.391	**0.001**
Naming	2.43 ± 0.67	2.31 ± 0.72	2.63 ± 0.52	2.62 ± 0.63	3.590	**0.014**
Attention	3.52 ± 1.56	4.23 ± 1.57	5.31 ± 0.93	4.92 ± 1.41	19.115	** < 0.001**
Language	2.25 ± 0.91	2.05 ± 1.01	2.43 ± 0.84	2.40 ± 0.91	2.190	**0.090**
Abstraction	1.25 ± 0.85	1.22 ± 0.75	1.28 ± 0.63	1.28 ± 0.66	0.113	0.952
Delayed memory	1.97 ± 1.64	2.68 ± 1.52	3.46 ± 1.40	3.17 ± 1.65	10.895	** < 0.001**
Orientation	4.57 ± 1.37	4.26 ± 1.70	5.81 ± 0.48	5.87 ± 0.44	29.577	** < 0.001**

Significant *p*-values are shown in bold cases

*SZ* Schizophrenia, *HC* Healthy controls, *BMI* Body mass index, *CPeq* Chlorpromazine-equivalent doses, *PANSS* Positive and Negative Syndrome Scale, *MoCA* Montreal Cogntive Assessment

^a^Age at the time of MR imaging

### GMV alterations

**Fig. 1:**
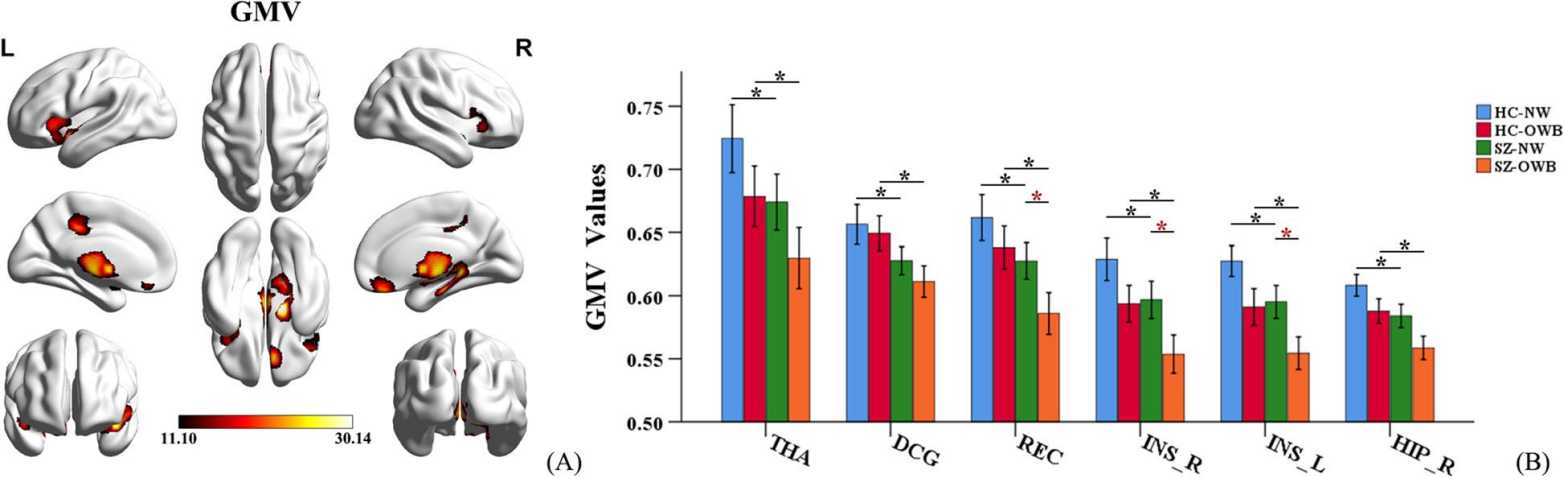
Brain regions showing significant main effects of diagnosis (*p* < 0.001, FWE corrected). **A** Results in the whole-brain VBM analysis: the red/orange indicates brain regions with lower GMV in SZ compared to HC group (SZ < HC), the color scales represent F-values. **B** A post hoc ROI analysis shows the pairwise comparisons in GMV, error bars indicate the standard error of the mean. *p < 0.001, FWE corrected. Abbreviations: SZ = schizophrenia; HC = healthy controls; L = left; R = right; HIP = hippocampus; INS = insula; REC = gyrus rectus; DCG = median cingulate and paracingulate gyri; THA = thalamus

There were significant main effects of diagnosis in the right hippocampus, bilateral insula, rectus, median cingulate/paracingulate gyri and thalamus, with reduced GMV in patients with SZ relative to HC (*p* < 0.001, FWE corrected) (Fig. 1A and Table 2). The post hoc analysis showed that both SZ-NW and SZ-OWB patients had significant GMV reduction in the aforementioned brain regions relative to that of the HC-NW and HC-OWB subjects, respectively (*p* < 0.001, FWE corrected). Meanwhile, SZ-OWB patients also showed significant GMV reduction in the bilateral insula and rectus relative to that of SZ-NW patients (*p* < 0.001, FWE corrected). No significant differences were observed in the aforementioned brain regions between HC-OWB and HC-NW participants (*p* > 0.001, FWE corrected) (Fig. 1B).

**Table 2:** Brain regions showing significant main effects of diagnosis and BMI in VBM analysis^*^

Brain region		Cluster size (voxels)	F value	MNI coordinate		
				**X**	**Y**	**Z**
**Main effects of diagnosis**						
Hippocampus	R	1307	30.1	16.5	-12	-12
Insula	L	2511	28.3	-45	4.5	-7.5
Insula	R	1031	19.1	54	21	1.5
Rectus	B	1110	22.9	7.5	37.5	-21
Cingulum-Mid	B	1224	21.1	-7.5	-31.5	42
Thalamus	B	1934	25.3	1.5	-18	-3
**Main effects of BMI**						
Amygdala	R	1982	24.8	37.5	-1.5	-5.5
Hippocampus	L	4339	27.2	-30	4.5	-34.5
Insula	L	1626	19.7	-24	22.5	-6
Insula	R	668	19.2	40.5	30	4.5
Lingual	L	1383	26.0	-24	-63	-1.5
Temporal_sup	R	1254	23.9	67.5	-13.5	4.5

*SZ* Schizophrenia, *OWB* Overweight or obesity, *L* Left, *R* Right, *B* Bilateral, *Cingulum-Mid* Median cingulate and paracingulate gyri, *Temporal_sup* Superior temporal gyrus, *MNI* Montréal neurological institute

^*^p < 0.001, FWE corrected

**Fig. 2:**
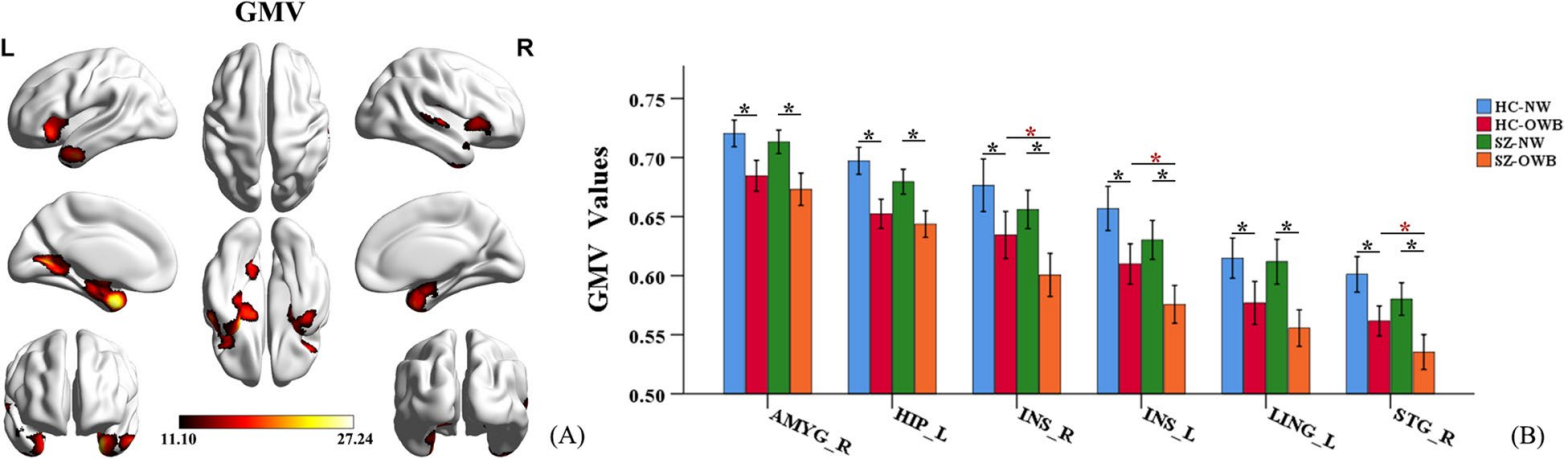
Brain regions showing significant main effects of BMI (*p* < 0.001, FWE corrected). **A** Results in the whole-brain VBM analysis: the red/orange indicates brain regions with lower GMV in OWB compared to NW group (OWB < NW), the color scales represent F-values. **B** A post hoc ROI analysis shows the pairwise comparisons in GMV, error bars indicate the standard error of the mean. *p < 0.001, FWE corrected. Abbreviations: OWB = overweight or obesity; NW = normal weight; L = left; R = right; AMYG = amygdala; HIP = hippocampus; INS = insula; LING = lingual gyrus; STG = superior temporal gyrus

In addition, there were significant main effects of BMI in the right amygdala, left hippocampus, bilateral insula, left lingual gyrus, and right superior temporal gyrus, with decreased GMV in OWB participants compared to NW individuals (*p* < 0.001, FWE corrected) (Fig. 2A and Table 2). The post hoc analyses showed that both the HC-OWB and SZ-OWB participants had significant GMV reduction in the aforementioned brain regions relative to that of the HC-NW and SZ-NW participants, respectively (*p* < 0.001, FWE corrected). Meanwhile, SZ-OWB patients also showed significant GMV reduction in the bilateral insula and right superior temporal gyrus relative to that of the HC-OWB patients (*p* < 0.001, FWE corrected). No significant differences were observed in the aforementioned brain regions between SZ-NW and HC-NW subjects (*p* > 0.001, FWE corrected) (Fig. 2B).

There were no significant diagnosis-by-BMI interaction effects in both whole-brain VBM analysis and multivariate analysis of covariance. The results of multivariate analysis of covariance are summarized in Table S3. Notably, the results showed that both SZ and OWB were additively associated with lower GMV in bilateral insula (Fig. S3).

### Partial correlation analysis

The relationship between GMV alterations and clinical symptoms is presented in Table 3. The brain regions displaying significant main effects of diagnosis are shown in Table 3a. In the SZ group, both the GMV values in the left insula and right insula were positively correlated with attention (*r* = 0.250, *p* = 0.003), and negatively correlated with PANSS negative scores (*r* = -0.184, *p* = 0.032; *r* = -0.214, *p* = 0.012, respectively). In addition, the GMV values in the rectus showed a positive relationship with abstraction (*r* = 0.210, *p* = 0.014), but a negative relationships with PANSS negative scores (*r* = -0.180, *p* = 0.035) and general psychopathology scores (*r* = -0.222, *p* = 0.009). Additionally, both the GMV in the thalamus and right hippocampus were positively correlated with visuospatial/constructional abilities (*r* = 0.197, *p* = 0.021and *r* = 0.191, *p* = 0.026, respectively), and the GMV in the thalamus was positively correlated with attention (*r* = 0.256, *p* = 0.003) in patients with SZ.

**Table 3:** Pearson’s partial correlations between brain GMV and clinical symptoms

**3a: Brain regions showing significant main effects of diagnosis**^**a**^						
	**Cingulum_Mid** **(r, *p*)**	**Insula-L** **(r, *p*)**	**Insula-R (r, *p*)**	**HIP-R** **(r, *p*)**	**Rectus** **(r, *p*)**	**Thalamus** **(r, *p*)**
**PANSS total**	-0.001, 0.991	-0.036, 0.678	-0.106, 0.218	0.005, 0.957	0.041, 0.634	-0.035, 0.685
PANSS positive	-0.061, 0.481	0.042, 0.627	-0.037, 0.671	-0.022, 0.801	-0.143, 0.094	0.046, 0.591
PANSS negative	-0.141, 0.100	-0.254, **0.003**	-0.301, **0.000**	-0.071, 0.412	-0.180, **0.035**	-0.139, 0.105
PANSS general psychopathology	0.097, 0.260	0.045, 0.600	0.002, 0.978	0.052, 0.548	0.222, **0.009**	-0.010, 0.904
**MoCA total**	0.082, 0.342	0.160, 0.062	0.192, **0.025**	0.142, 0.099	0.114, 0.184	0.137, 0.110
Visuospatial/constructional	0.044, 0.611	0.148, 0.084	0.163, 0.057	0.191, **0.026**	0.098, 0.253	0.197, **0.021**
Naming	-0.012, 0.890	0.039, 0.654	0.015, 0.866	0.087, 0.310	0.031, 0.723	0.090, 0.294
Attention	0.154, 0.072	0.250, **0.003**	0.250, **0.003**	0.157, 0.067	0.151, 0.078	0.256, **0.003**
Language	0.006, 0.948	0.053, 0.540	0.112, 0.193	0.044,0.609	0.009, 0.917	-0.014, 0.874
Abstraction	-0.098, 0.252	-0.019, 0.823	0.015, 0.865	-0.045,0.599	0.210, **0.014**	-0.074, 0.391
Delayed memory	-0.008, 0.929	0.116, 0.177	0.114, 0.184	0.102, 0.234	-0.025, 0.770	0.026, 0.759
Orientation	0.117, 0.174	-0.015, 0.864	0.059, 0.496	0.069, 0.420	0.024, 0.777	0.053, 0.539
**3b: Brain regions showing significant main effects of BMI**^**b**^						
	**HIP_L** **(r, *p*)**	**Insula-L** **(r, *p*)**	**Insula-R (r, *p*)**	**LING_L** **(r, *p*)**	**AMYG_R** **(r, *p*)**	**STG_R** **(r, *p*)**
**MoCA total**	0.012, 0.900	0.180, 0.052	0.118, 0.201	0.013, 0.889	0.362, **0.000**	0.360, **0.000**
Visuospatial/constructional	0.050, 0.591	0.058, 0.532	0.038, 0.684	-0.151,0.103	0.134, 0.149	0.184, **0.046**
Naming	-0.106, 0.254	-0.028, 0.762	-0.101, 0.276	0.072, 0.436	0.284, **0.002**	0.087, 0.347
Attention	0.066, 0.479	0.253, **0.006**	0.260, **0.004**	0.158, 0.087	0.287, **0.002**	0.302, **0.001**
Language	-0.143, 0.123	0.090, 0.332	-0.059, 0.529	-0.168, 0.069	0.087, 0.351	0.116, 0.212
Abstraction	-0.043, 0.643	-0.027, 0.768	-0.059, 0.523	-0.068, 0.465	0.066, 0.478	0.099, 0.287
Delayed memory	0.019, 0.836	0.117, 0.206	0.100, 0.284	0.029, 0.758	0.307, **0.001**	0.219, **0.017**
Orientation	0.059, 0.523	0.081, 0.385	0.049, 0.595	0.105, 0.256	0.208, **0.024**	0.294, **0.001**

Significant *p*-values are shown in bold cases

*SZ* Schizophrenia, *OWB* Overweight or obesity, *PANSS* Positive and Negative Syndrome Scale, *MoCA* Montreal Cogntive Assessment, *L* Left, *R* Right, *HIP* Hippocampus, *AMYG* Amygdala, *LING* Lingual gyrus, *STG* Superior temporal gyrus, *BMI* Body mass index, *ICV* Total intracranial volume

^a^Correlations between brain GMV and clinical symptoms (PANSS and MoCA) in the SZ group, controlling for age, sex, education, illness duration, BMI and ICV

^b^Correlations between brain GMV and clinical symptoms (MoCA) in OWB group, controlling for age, sex, education, BMI and ICV

The brain regions that showed significant main effects of BMI are shown in Table 3b. Among the participants with overweight/obesity, both the GMV values in the left insula and right insula were positively correlated with attention (*r* = 0.253, *p* = 0.006; *r* = 0.260, *p* = 0.004, respectively), while the GMV alterations in the right amygdala and superior temporal gyrus had positive relationships with both the MoCA total and subscale scores (including visuospatial/constructional abilities, naming, attention, delayed memory, and orientation) (all *p* < 0.05).

### Mediation analysis

**Fig. 3:**
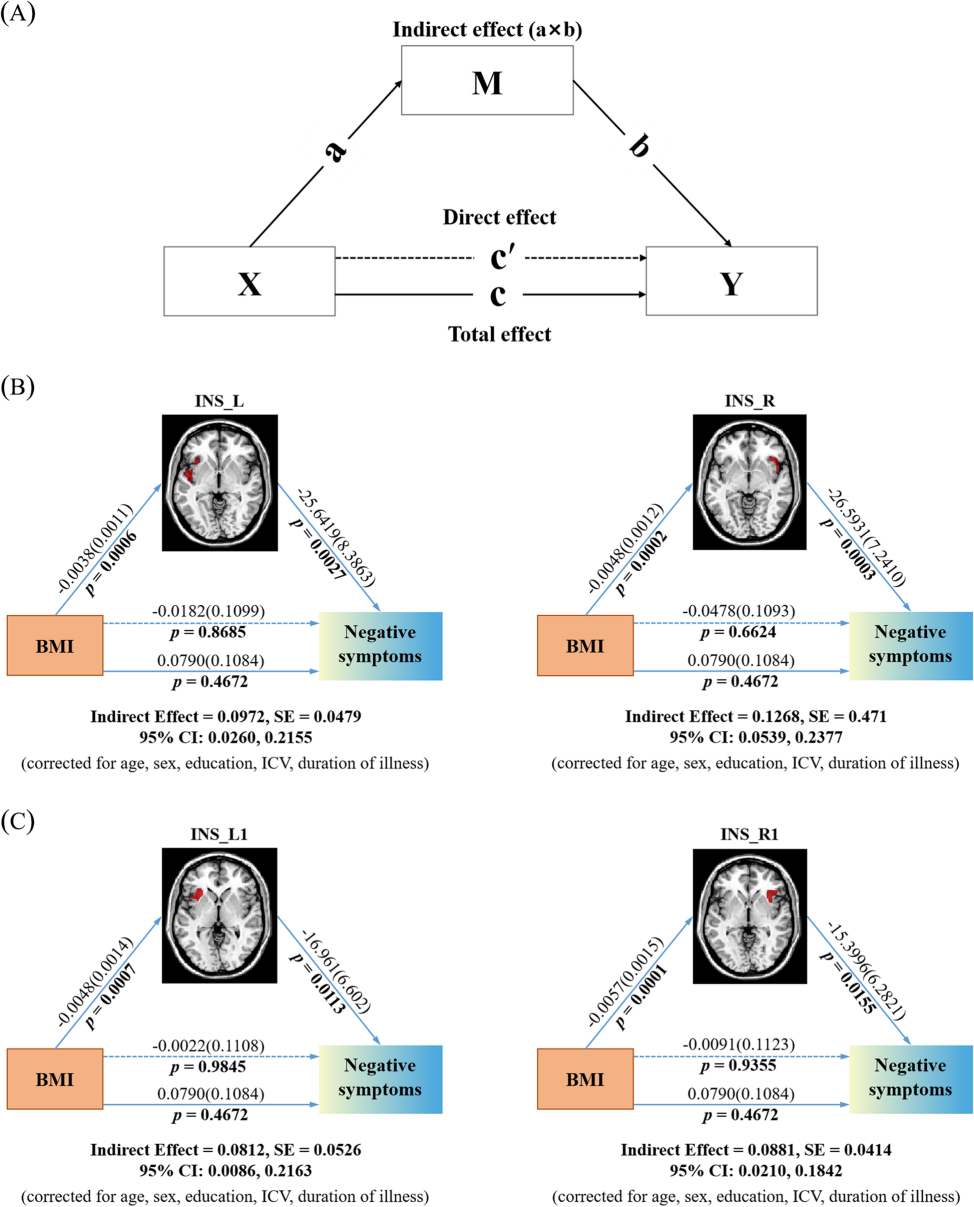
Mediation analysis testing whether GMV in bilateral insula mediates the relationship between BMI and negative symptoms in SZ. The age, sex, education, ICV, and duration of illness were included as covariates. **A** Standard 3-variable path model of mediation analysis. Path c is the total effect of X on Y; path c′ is the direct effect of X on Y after controlling for M; the product of paths a and b (a × b) is the indirect effect of X on Y through M. **B** Results from the bilateral insula identified in main effects of diagnosis: INS_L (left) and INS_R (right). **C** Results from the bilateral insula identified in main effects of BMI: INS_L1 (left) and INS_R1 (right). Unstandardized path coefficients are displayed along with standard errors in parentheses, and significance levels (ie, *p* value) underneath them. The total effects, direct effects, and indirect effects were considered significant when the *p* < 0.05 or 95% CIs did not contain zero. *Note*: GMV, gray matter volume; INS, insula; L, left; R, right; SZ, schizophrenia; OWB, overweight/obesity; ICV, total intracranial volume; BMI, body mass index; SE, standard errors; CIs, confidence intervals

In the SZ group, both the BMI and negative symptom scores showed a negative relationship with the bilateral insula (Figs. S1 and S2). To determine whether the effect of BMI on negative symptom was mediated by altered GMV in the bilateral insula, a mediation analysis was conducted in the SZ group. Mediation analysis revealed an indirect effect of BMI on negative symptom via GMV alterations in the bilateral insula (Fig. 3); however, the total and direct effects of BMI on negative symptom were not significant, indicating that inhibitory effects existed [26], and the association between BMI and negative symptom became statistically significant only when the GMV in the bilateral insula was included in the models. Hence, the lower GMV in the bilateral insula mediates the effect of higher BMI on negative symptom severity.

Specifically, for the bilateral insula identified in main effects of diagnosis, BMI had significant indirect effects on negative symptom severity via GMV in left the insula (a × b = 0.0972, 95% CI: 0.0260, 0.2155) or right insula (a × b = 0.1268, 95% CI: 0.0539, 0.2377) and had no significant direct effect on negative symptom severity (c′ = -0.0182, *p* = 0.8685; c′ = -0.0478, *p* = 0.6624; respectively) (Fig. 3B). In addition, similar results were found for the bilateral insula identified in main effects of BMI, BMI had significant indirect effects on negative symptom severity via GMV in the left insula (a × b = 0.0812, 95% CI: 0.0086, 0.2163) or right insula (a × b = 0.0881, 95% CI: 0.0210, 0.1842) and had no significant direct effect on negative symptom severity (c′ = -0.0022, *p* = 9845; c′ = -0.0091, *p* = 0.9355; respectively) (Fig. 3C).

## Discussion

In this study, we investigated the impact of the diagnosis and BMI on brain GMV, and we demonstrated that lower GMV in the right hippocampus, bilateral insula, rectus, median cingulate/paracingulate gyri, and thalamus were associated with SZ, while lower GMV in the right amygdala, left hippocampus, bilateral insula, left lingual gyrus, and right superior temporal gyrus were associated with being overweight/obesity independent of SZ diagnosis. There were no significant diagnosis-by-BMI interaction effects in this study, but the results showed that both SZ and OWB were additively associated with lower GMV in the bilateral insula. In SZ, mediation analysis revealed an indirect effect of BMI on negative symptom via GMV reduction in the bilateral insula. Altogether, these findings provide further evidence that higher BMI is associated with lower GMV, which could increase the risk of unfavorable disease course in SZ.

We identified significant main effects of SZ diagnosis in the frontal, temporal, insular, and thalamus regions, which are considered the most replicated regions observed in SZ [3, 4]. The rectus and median cingulate/paracingulate gyri are integral parts of the frontal cortex [27], structural abnormalities in these two regions are responsible for impulsive behavior in SZ [28, 29]. Additionally, both the rectus (part of the orbitofrontal cortex proper) and insula are major components of the reward circuit [30]. Disrupted reward circuit has been proposed as the neuropathological basis of negative symptoms, manifested as apathy and social withdrawal in SZ [31]. This hypothesis is further supported by findings from the present study that lower GMV in rectus and insula were related to worsen negative symptom in patients with SZ. However, it is worth noting that insula abnormalities have also been reported to be associated with positive symptoms [32], so future studies may need to disentangle (or map) the abnormal structure, function and connectivity of insular subregions to symptoms, behavior and physiology. The thalamus is a major information hub implicated in multiple conscious integration processes, including cognition, emotion, and sensorimotor information processing [33, 34]. In support of this, our study also showed that a lower GMV in the thalamus was associated with cognitive decline (including visuospatial/constructional abilities and attention) in patients with SZ.

Moreover, the current study also identified lower GMV in temporal and insular regions in overweight/obese subjects, regardless of the SZ diagnosis. Temporal regions are associated with many cognitive processes [35, 36]. Substantial evidence suggests that gray matter atrophy in the temporal regions is associated with poor cognitive performance in obesity [15, 37], which is consistent with findings of our study that GMV loss in the amygdala, hippocampus and superior temporal gyrus were related with multiple cognitive impairments. Further, hippocampal-dependent learning and memory mechanisms have significant impacts on the regulation of food intake, mainly through the integration of external sensory information (olfactory and visuospatial) and internal cues (gustatory, endocrine, and gastrointestinal interoceptive stimuli) [38, 39]. In support are findings that hippocampal lesions lead to increased food intake and subsequent obesity in both animal and human studies [40, 41]. Similarly, the amygdala is known for detecting salient stimuli [42], which shows consistent functional activation in response to food-related stimuli and has consistently been observed in research on appetitive behavior [43, 44]. In addition, the insular cortex is predominantly involved in empathy, self-awareness, and interoception [45]. Previous studies have detected activation of the insula using balloon distention to mimic gastric dilation, thus indicating its role in the perception of fullness [46]. There is also evidence that blunt self-awareness of satiety signals may cause obese individuals to increase their food intake in response to interoceptive cues [47]. Along with these findings from previous and our study, we suppose that the brain structural deficits in the temporal and insular regions may underlie the dysfunction of appetite regulation in overweight/obese subjects.

Another obesity-affected region in the present study was the left lingual gyrus, a key part of visual information processing [48]. Previous studies have detected robust activation of the occipital cortex in response to visually presented food cues [49]. Recently, Stopyra et al. [50] revealed that increased activation of the lingual gyrus is strongly associated with a decrease in food craving. In the field of obesity, researchers have revealed a reduced activation of the occipital cortex in obese individuals [51]. Based on these evidence, it is possible that GMV loss in the left lingual gyrus may underlie the reduced brain activity in this area, thus leading to a stronger increase in food craving in overweight/obese subjects.

In the present study, most of the SZ-related and overweight/obesity-related brain regions were identified in the limbic regions associated with reward information processing, i.e., bilateral insula (sensory processing), amygdala-hippocampus (reward learning and memory), rectus and median cingulate/paracingulate gyri (reward value appraisal, executive control and decision-making) [27, 32, 42]. Indeed, appropriate responses to external salient stimuli, especially rewarding cues, require constant updating and learning to adjust behaviors according to new data [52]. This process requires coordination of all the above brain regions, thus structural deficits in any of these regions may lead to abnormalities in expressed behaviors. In line with this, theories of SZ indicate that dysfunction of the reward circuit is associated with negative symptom, primarily through disruption of reward processing and promotion of false integration of the inner sensory perception with salient stimuli [30]. Similarly, theories of obesity suggest that both hyporesponsivity to rewards and high levels of trait reward sensitivity cause individuals to consume more high-fat and high-calorie foods, thus increasing their vulnerability to overeating and obesity [53]. These theories support the role of neural reward circuits in both SZ and obesity. This similarity in the GMV deficit pattern may also serve as a potential explanation for the high prevalence of overweight or obesity in patients with SZ.

Notably, despite the relatively large sample size of this study (*N* = 250), we were not powered to detect a significant interaction effect in a whole-brain analysis having to correct for multiple (voxel-by-voxel) comparisons. However, the results did show that both SZ and overweight/obesity were additively related to lower GMV in bilateral insula. As a paralimbic structure, the insula has been implicated in a wide range of conditions and behaviors, from interoception of body sensations and movement to selective attention and salience detection [45]. GMV reduction in this region has been reported to be linked to negative symptom (e.g., anhedonia and amotivation) in SZ, which may be related to deficient insular detection of salience in reward processing [54]. The insula is a key node of the reward circuit, which is also involved in reward-related eating behaviors such as craving, feeding, and satiety [55]. Blunted activation of the insula is thought to result in overeating and subsequent obesity [56]. Conversely, overeating or obesity can further reduce the neural response to food cues, especially in hypothalamus and insular cortex, thereby leading to a vicious cycle [57, 58]. Thus, the common GMV deficits in bilateral insula may represent a key intersection point for both SZ and overweight/obesity.

Furthermore, the bilateral insula identified in both SZ and OWB was associated with attention deficits in the respective illnesses. In addition, mediation analysis revealed an indirect effect of BMI on negative symptom via GMV alterations in bilateral insula. These results are highly clinically relevant, as both SZ and OWB are responsible for worse cognitive performance and comorbidity with overweight/obesity often contribute to worse psychiatric outcomes in patients with SZ [59, 60]. As such, our findings suggest a critical role of the bilateral insula in the neuropathology of SZ as a potential adverse consequence of overweight/obesity that might contribute to an unfavorable course of disease. Moreover, both negative symptom and cognitive deficits tend to be resistant to antipsychotic treatments, and the presence of negative symptom early in the illness is closely related to a worsening course of the disease and greater lifelong disability [61]. Thus, identifying the mechanisms underlying such brain structural deficits in the bilateral insula may yield preventative and therapeutic targets for both SZ and OWB. Of relevance, longitudinal studies in the general population have shown that overweight/obesity-related brain structural alterations might be preventable or even reversible with lifestyle/medication/surgical interventions focused on weight management, particularly in teenagers and young adults [62–64]. Consistent with this evidence, lifestyle interventions for weight loss have shown positive effects in improving functional outcomes in recurrent depression [65], which are related to increases in brain volume [66, 67]. Therefore, efforts to foster weight loss may help address some common but currently intractable clinical outcomes such as negative symptom and attention deficits in SZ.

Finally, a previous study with a similar design reported that both SZ and overweight/obesity were associated with overlapped GMV alterations in the cerebellum [20], a region deeply connected to the cerebrum and had been proven to have potential functions in cognitive and mood regulation [68]. However, our study failed to identify similar results in the cerebellum. One explanation is that the effects of SZ on brain structural alterations may have been confounded by prolonged exposure to antipsychotics in our study, since long-term antipsychotic treatment have potential effects on brain structure [69]. Other factors, such as differences in the underlying methodology, as well as differences in the power and clinical characteristics of the study samples, might have also contributed to this inconsistent finding. Therefore, future studies are required to substantiate these findings in unmedicated patients with first-episode psychosis.

### Limitations

This study has some limitations. First, this study had a cross-sectional design, which precluded causal inferences, thus longitudinal design is needed to determine whether GMV loss occurred before or after the weight gain abnormalities in patients with SZ. Second, the results of the mediation analysis should be interpreted with caution, as it is challenging to discuss causality in a mediation analysis. Future longitudinal research should be conducted to clarify the causal relationship between BMI and negative symptoms. Third, we did not collect information on the factors that might influence the correlation between BMI and brain structure, such as chronic stressors, diet, and exercise. Future studies will benefit from this consideration. Finally, the SZ participants in this study mainly consisted of chronic patients taking medications, thus failing to eliminate the confounders of illness chronicity and prolonged exposure to antipsychotics on brain alterations. However, to minimize the effect of these factors, the two SZ groups were matched for duration of illness and use of antipsychotic medications. To further increase homogeneity, we did not include participants with metabolic comorbidities, such as diabetes.

## Conclusion

In summary, the present study provides further evidence that both SZ and OWB adversely affect similar brain regions associated with reward processing, in which the common brain structural deficits in the bilateral insula may represent a key intersecting point. This similarity in the morphometric deficit pattern may serve as a potential explanation for the higher prevalence of overweight/obesity in patients with SZ. Moreover, this study provides preliminary support for the role of the bilateral insula in mediating the indirect effect of BMI on negative symptoms, which further indicates a critical role of the bilateral insula in the neuropathology of SZ. Taken together, these findings suggest that higher BMI is associated with lower GMV, and more consideration should be given to the insula pathophysiology, which may serve as a potential novel target for the treatment of both SZ and OWB.

## Supplementary Information

**Additional file 1: Fig. S1.** Partial correlation analysis between BMI and bilateral insula in SZ group, controlling for sex, age and education. **Fig. S2.** Partial correction analysis between negative symptom and bilateral insula in SZ group, controlling for sex, age and education. **Fig. S3.** Results of bilateral insula in the whole-brain VBM analyses. Red indicates the bilateral insula in the main effects of diagnosis (SZ<HC); Yellow indicates bilateral insula in the main effects of BMI (OWB<NW); color between yellow and red indicate overlapping brain regions (orange). Results are displayed superimposed on the ch2bet template. **Fig. S4.** Mediation analysis testing whether GMV in bilateral insula mediates the relationship between BMI and negative symptoms in SZ. The age, sex, education, and ICV were included as covariates. **Table S1.** Demographic and clinical characteristics for SZ patients. **Table S2.** Demographic and clinical characteristics for HC participants. **Table S3.** The multivariate analysis of covariance. **Table S4. **Pearson’s partial correlations between brain GMV and clinical symptoms, controlling for age, sex, education, BMI and ICV.

## Data Availability

The datasets generated in this study are available from the corresponding author on reasonable request.
