# Supplementary figures and images for: Gray matter reduction in bilateral insula mediating adverse psychiatric effects of body mass index in schizophrenia
Source: BMC Psychiatry. 2022 Oct 11;22:639. doi: 10.1186/s12888-022-04285-4 (PMC9552355; doi:10.1186/s12888-022-04285-4)

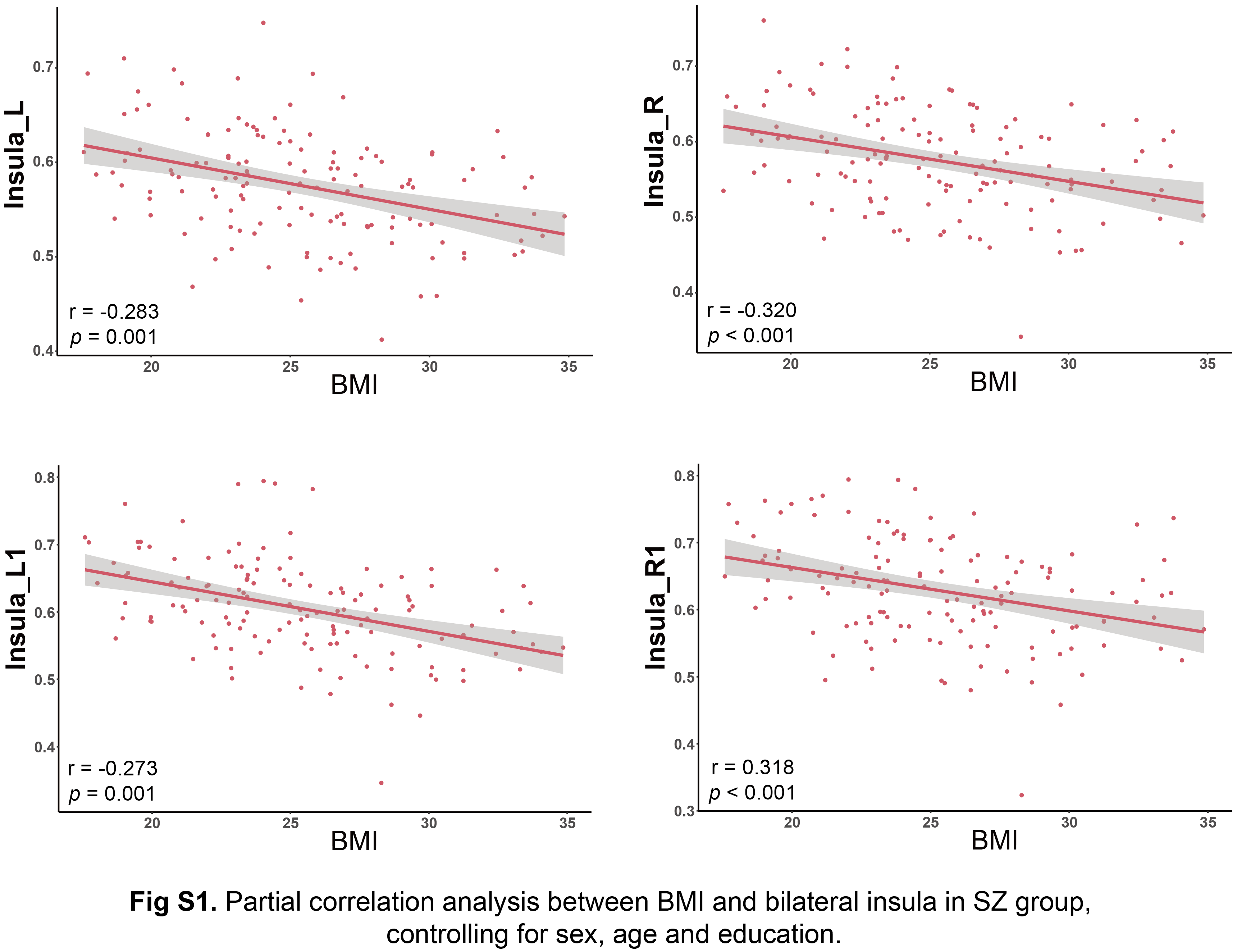

Supplement: Supplementary file 1 — Additional file 1: Fig. S1. Partial correlation analysis between BMI and bilateral insula in SZ group, controlling for sex, age and education. Fig. S2. Partial correction analysis between negative symptom and bilateral insula in SZ group, controlling for sex, age and education. Fig. S3. Results of bilateral insula in the whole-brain VBM analyses. Red indicates the bilateral insula in the main effects of diagnosis (SZ<HC); Yellow indicates bilateral insula in the main effects of BMI (OWB<NW); color between yellow and red indicate overlapping brain regions (orange). Results are displayed superimposed on the ch2bet template. Fig. S4. Mediation analysis testing whether GMV in bilateral insula mediates the relationship between BMI and negative symptoms in SZ. The age, sex, education, and ICV were included as covariates. Table S1. Demographic and clinical characteristics for SZ patients. Table S2. Demographic and clinical characteristics for HC participants. Table S3. The multivariate analysis of covariance. Table S4. Pearson’s partial correlations between brain GMV and clinical symptoms, controlling for age, sex, education, BMI and ICV. [file 12888_2022_4285_MOESM1_ESM.zip › Supplementary_Material/Fig S1.tif]

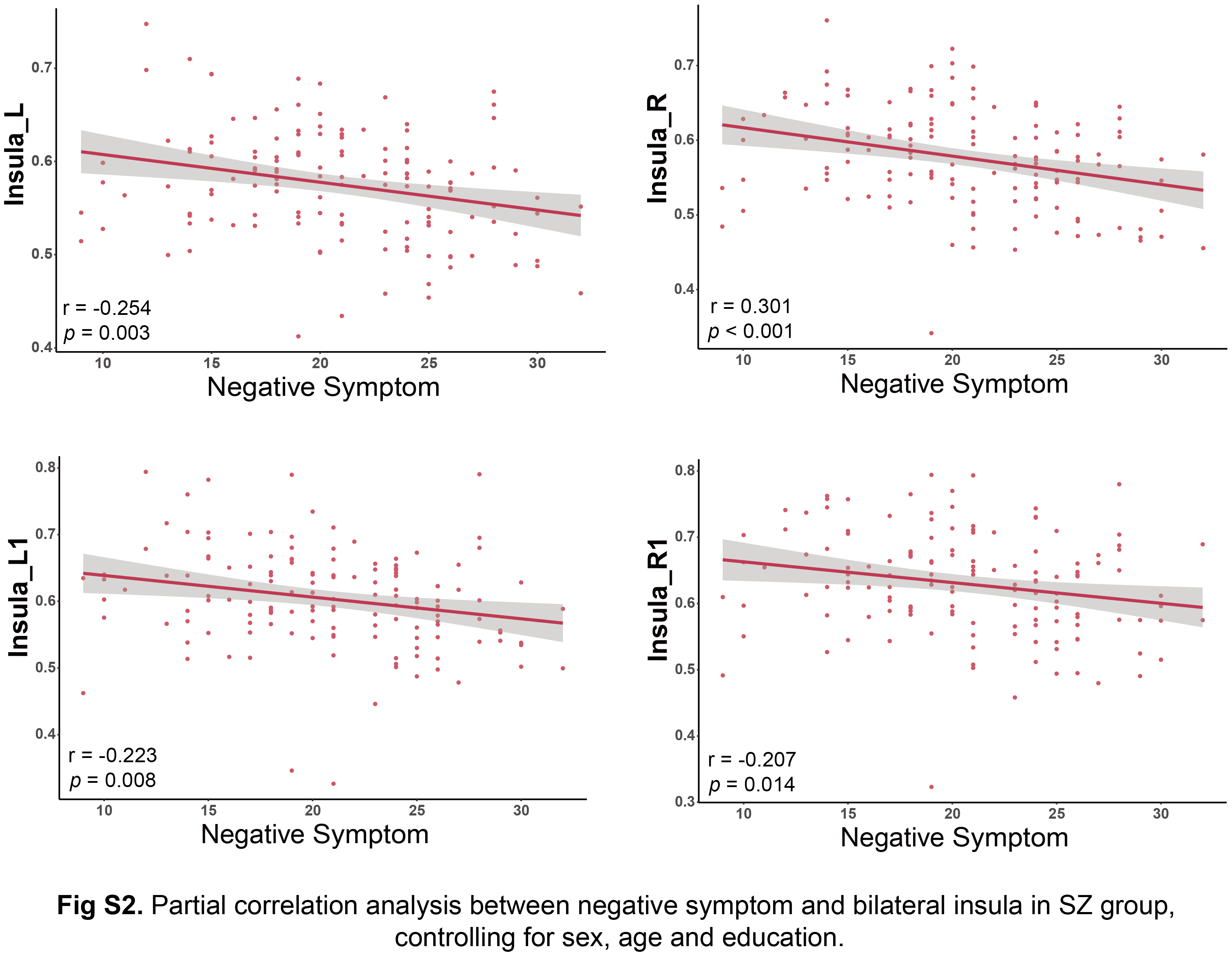

Supplement: Supplementary file 1 — Additional file 1: Fig. S1. Partial correlation analysis between BMI and bilateral insula in SZ group, controlling for sex, age and education. Fig. S2. Partial correction analysis between negative symptom and bilateral insula in SZ group, controlling for sex, age and education. Fig. S3. Results of bilateral insula in the whole-brain VBM analyses. Red indicates the bilateral insula in the main effects of diagnosis (SZ<HC); Yellow indicates bilateral insula in the main effects of BMI (OWB<NW); color between yellow and red indicate overlapping brain regions (orange). Results are displayed superimposed on the ch2bet template. Fig. S4. Mediation analysis testing whether GMV in bilateral insula mediates the relationship between BMI and negative symptoms in SZ. The age, sex, education, and ICV were included as covariates. Table S1. Demographic and clinical characteristics for SZ patients. Table S2. Demographic and clinical characteristics for HC participants. Table S3. The multivariate analysis of covariance. Table S4. Pearson’s partial correlations between brain GMV and clinical symptoms, controlling for age, sex, education, BMI and ICV. [file 12888_2022_4285_MOESM1_ESM.zip › Supplementary_Material/Fig S2.tif]

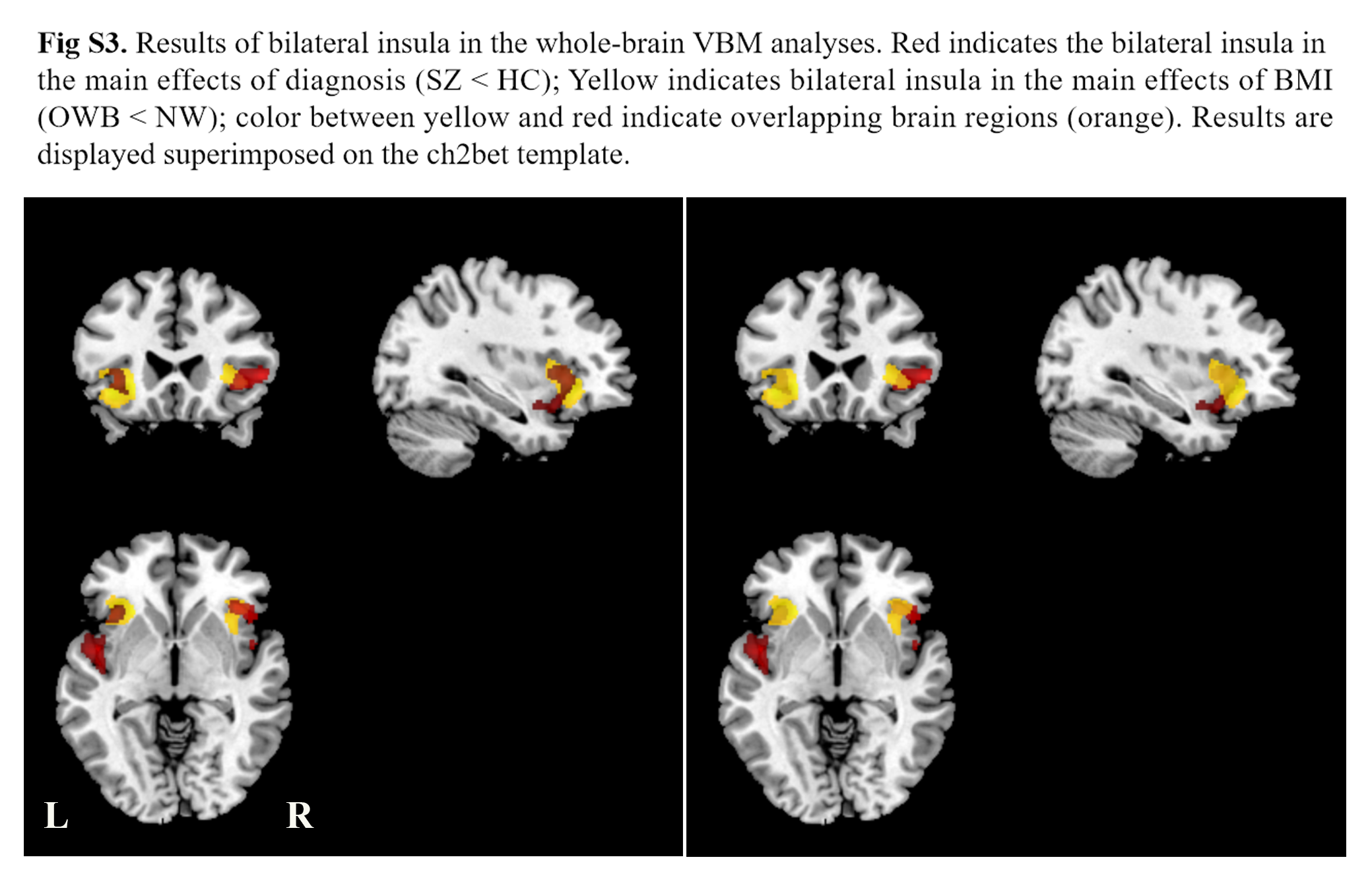

Supplement: Supplementary file 1 — Additional file 1: Fig. S1. Partial correlation analysis between BMI and bilateral insula in SZ group, controlling for sex, age and education. Fig. S2. Partial correction analysis between negative symptom and bilateral insula in SZ group, controlling for sex, age and education. Fig. S3. Results of bilateral insula in the whole-brain VBM analyses. Red indicates the bilateral insula in the main effects of diagnosis (SZ<HC); Yellow indicates bilateral insula in the main effects of BMI (OWB<NW); color between yellow and red indicate overlapping brain regions (orange). Results are displayed superimposed on the ch2bet template. Fig. S4. Mediation analysis testing whether GMV in bilateral insula mediates the relationship between BMI and negative symptoms in SZ. The age, sex, education, and ICV were included as covariates. Table S1. Demographic and clinical characteristics for SZ patients. Table S2. Demographic and clinical characteristics for HC participants. Table S3. The multivariate analysis of covariance. Table S4. Pearson’s partial correlations between brain GMV and clinical symptoms, controlling for age, sex, education, BMI and ICV. [file 12888_2022_4285_MOESM1_ESM.zip › Supplementary_Material/Fig S3.tif]

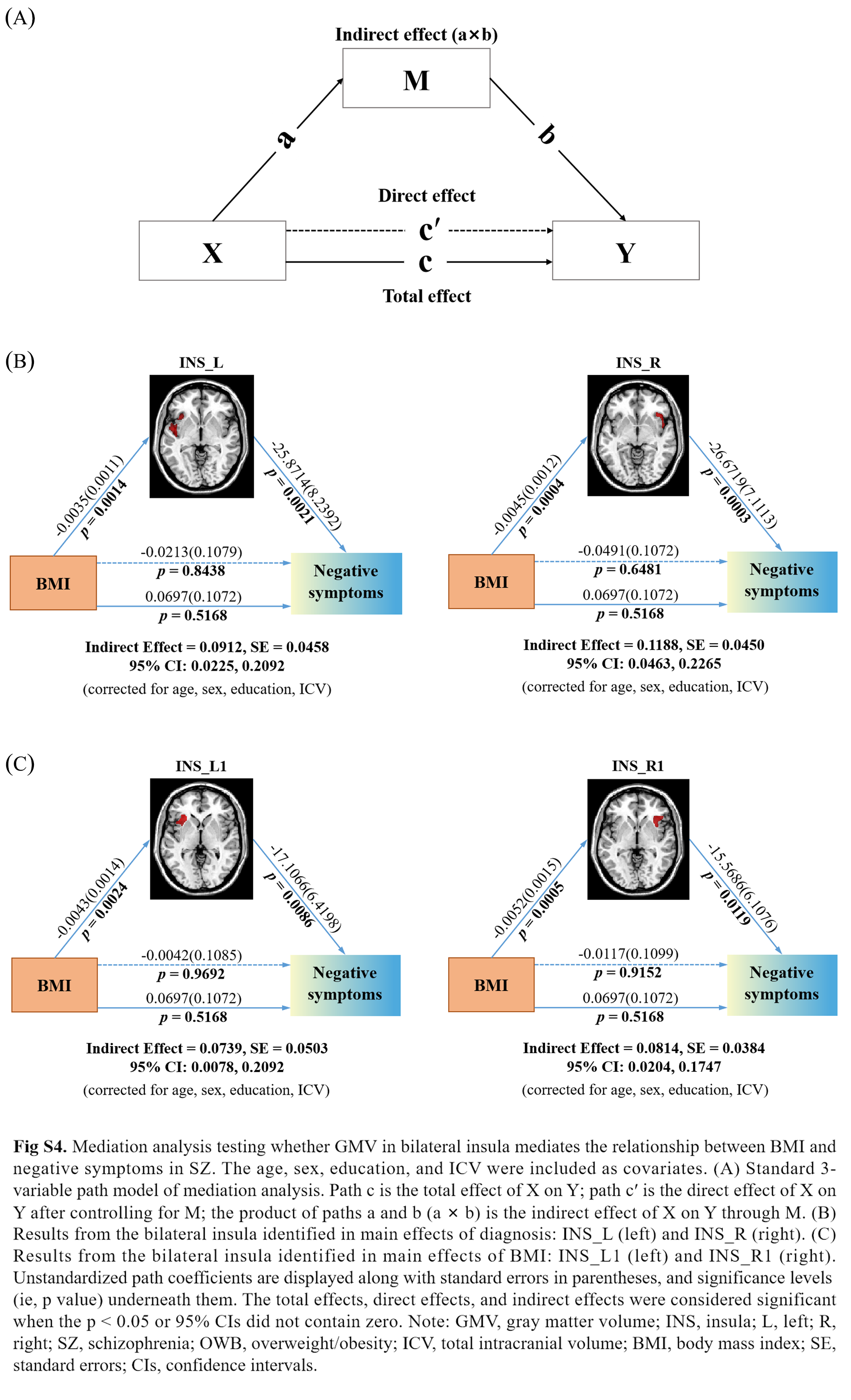

Supplement: Supplementary file 1 — Additional file 1: Fig. S1. Partial correlation analysis between BMI and bilateral insula in SZ group, controlling for sex, age and education. Fig. S2. Partial correction analysis between negative symptom and bilateral insula in SZ group, controlling for sex, age and education. Fig. S3. Results of bilateral insula in the whole-brain VBM analyses. Red indicates the bilateral insula in the main effects of diagnosis (SZ<HC); Yellow indicates bilateral insula in the main effects of BMI (OWB<NW); color between yellow and red indicate overlapping brain regions (orange). Results are displayed superimposed on the ch2bet template. Fig. S4. Mediation analysis testing whether GMV in bilateral insula mediates the relationship between BMI and negative symptoms in SZ. The age, sex, education, and ICV were included as covariates. Table S1. Demographic and clinical characteristics for SZ patients. Table S2. Demographic and clinical characteristics for HC participants. Table S3. The multivariate analysis of covariance. Table S4. Pearson’s partial correlations between brain GMV and clinical symptoms, controlling for age, sex, education, BMI and ICV. [file 12888_2022_4285_MOESM1_ESM.zip › Supplementary_Material/Fig S4.tif]
